# Supplementary material for: Cryo-EM structures of thylakoid-located voltage-dependent chloride channel VCCN1
Source: Nat Commun. 2022 May 6;13:2505. doi: 10.1038/s41467-022-30292-w (PMC9076864; doi:10.1038/s41467-022-30292-w)

Supplementary Information

Cryo-EM structures of thylakoid-located voltage-dependent chloride channel VCCN1

Hagino et al.

Supplementary Figures 1-16
Supplementary Table 1

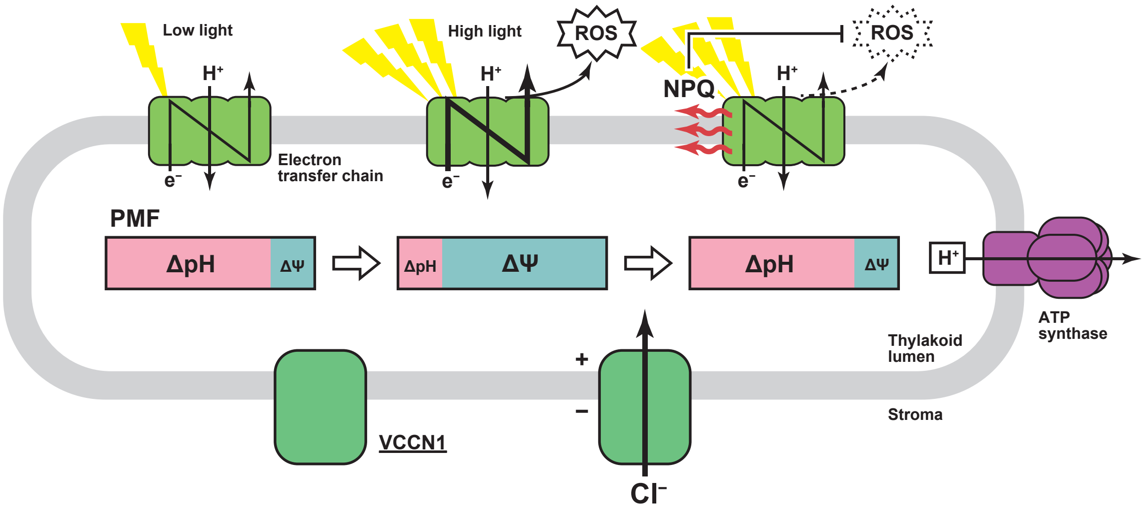

Supplementary Fig. 1 | Function of VCCN1 in tuning of photosynthesis.

Schematic diagram of the PMF partitioning by VCCN1 for adapting photosynthesis to high levels of light. In photosynthesis, the PMF for ATP synthesis is stored mainly as ΔpH. VCCN1 is rapidly activated and induces Cl^- influx in response to the change of ΔΨ, caused by the transition from low to high light. Cl^- influx dissipates ΔΨ and maintains the high contribution of ΔpH, which activates NPQ for suppressing the generation of ROS. The bar graphs of PMF represent only the ratio of ΔpH to ΔΨ, and do not depict the absolute amounts.

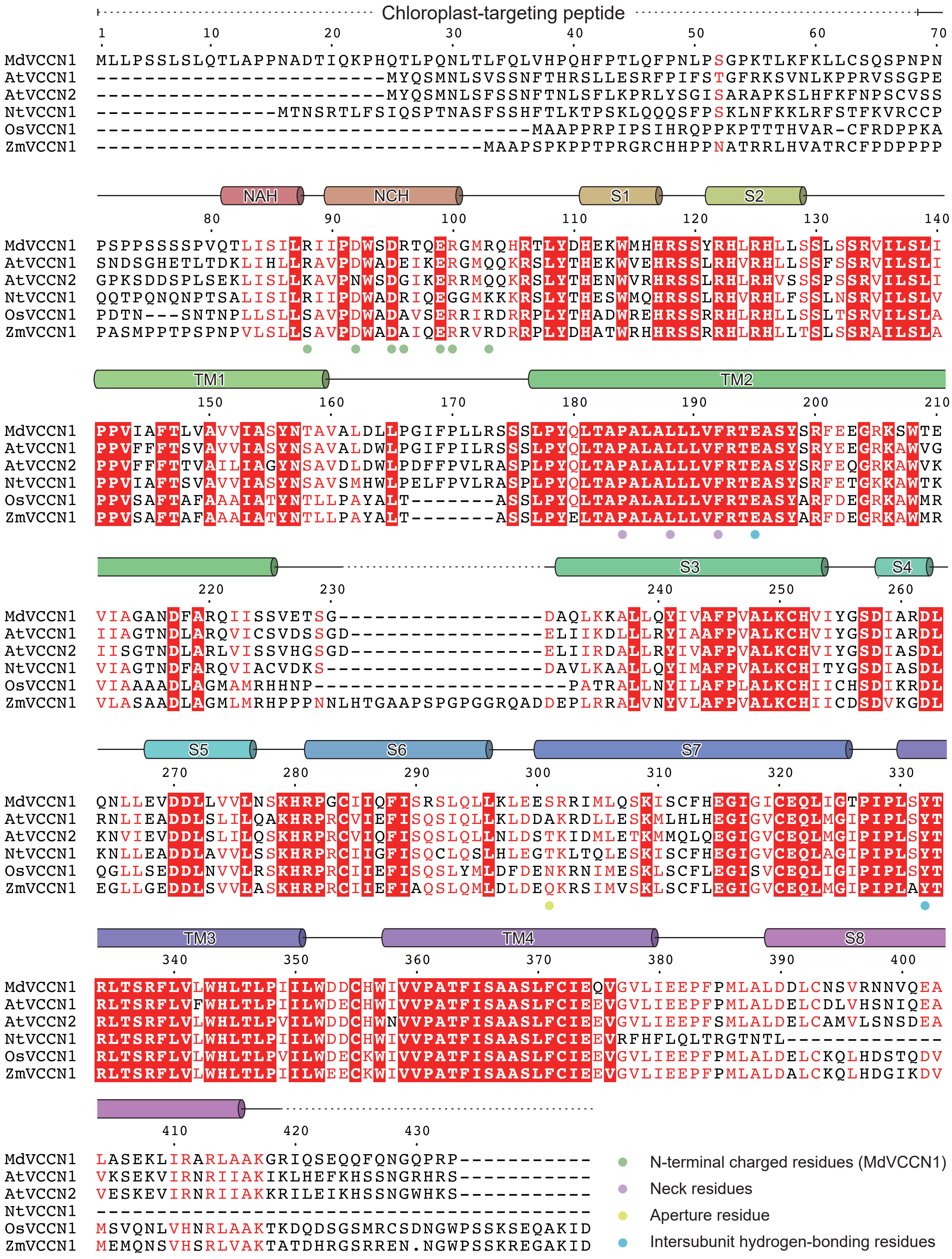

Supplementary Fig. 2 | Multiple sequence alignment of VCCN1 homologues.

Amino acid sequence alignment of *Malus domestica* VCCN1 (MdVCCN1 [NCBI accession number: XP_028961536]), *Arabidopsis thaliana* VCCN1 (AtVCCN1 [UniProt: Q9M2D2]), *Arabidopsis thaliana* VCCN2 (AtVCCN2 [UniProt: O80832]), *Nicotiana tabacum* VCCN1 (NtVCCN1 [NCBI accession number: XP_016458251.1]), *Oryza sativa* VCCN1 (OsVCCN1 [NCBI accession number: XP_015628296]) and *Zea mays* VCCN1 (ZmVCCN1 [GenBank: PWZ53889]). The amino acid sequences were aligned with ClustalW[47] and are shown by ESPript3[48]. The secondary structures of MdVCCN1 are illustrated above the sequence.

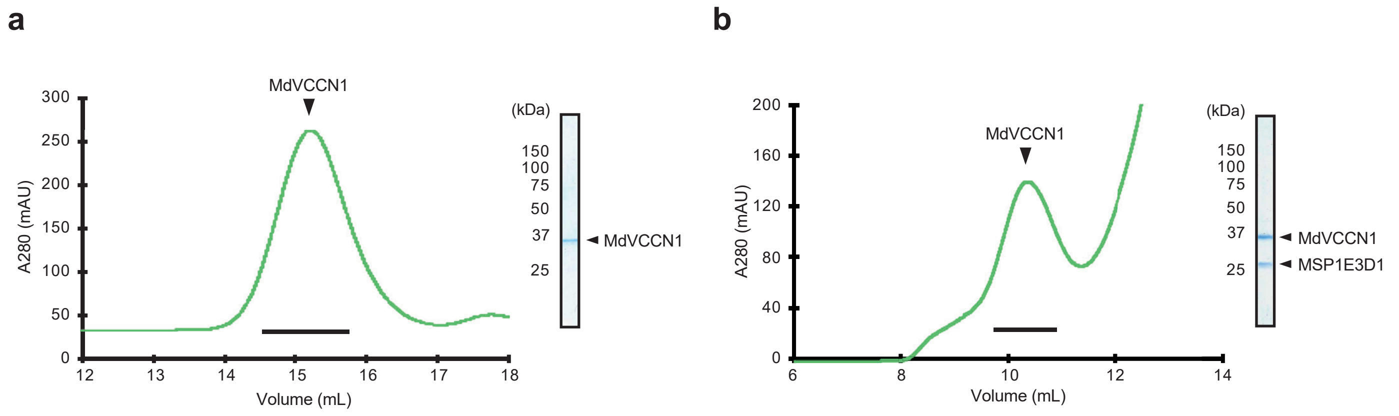

Supplementary Fig. 3 | Purification profiles of MdVCCN1.

a, SEC chromatogram using a Superose 6 Increase 10/300 column (left) and SDS-PAGE gel image (right) of MdVCCN1 in GDN. The experiments were repeated twice independently, with similar results. **b**, SEC chromatogram using a Superdex 200 10/300 Increase column (left) and SDS-PAGE gel image (right) of MdVCCN1 in nanodiscs. In both panels, the black bar represents the area of collected fractions. The experiment was performed once.

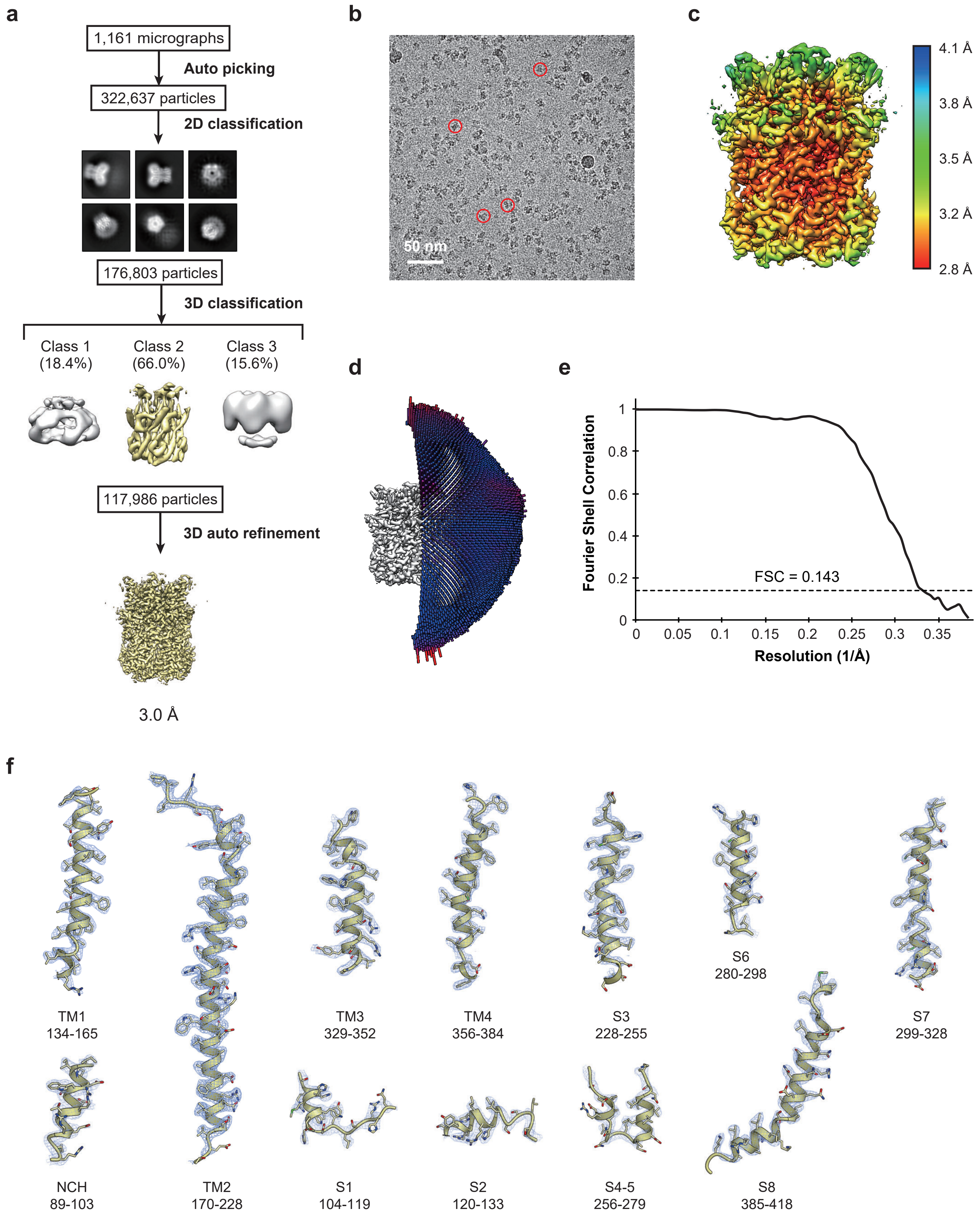

Supplementary Fig. 4 | Single-particle analysis of MdVCCN1 in GDN.

a, Workflow of the single-particle analysis of MdVCCN1 in GDN. **b**, Representative cryo-EM micrograph of MdVCCN1 in GDN. The red circles indicate individual particles. The white scale bar represents 50 nm. The data collection was performed once. **c**, Local resolution map of MdVCCN1 in GDN. **d**, Angular distribution plot of particles included in the final 3D reconstruction of MdVCCN1 in GDN, with imposed C5 symmetry. **e**, Fourier shell correlation (FSC) curve of the final map of MdVCCN1 in GDN. The horizontal dashed line represents the criterion of FSC = 0.143. **f**, Fragmented models of MdVCCN1 in GDN with corresponding cryo-EM density maps.

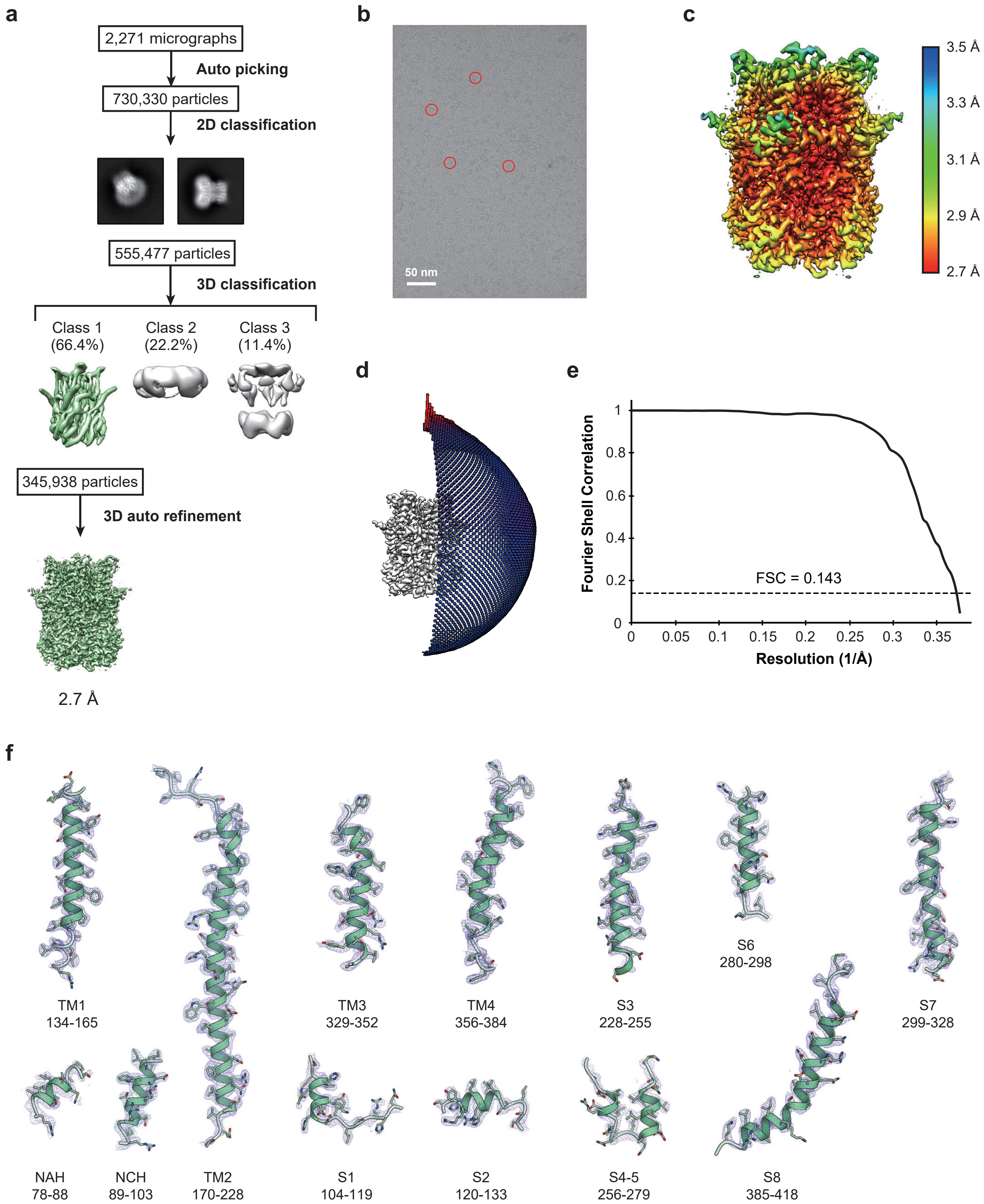

Supplementary Fig. 5 | Single-particle analysis of MdVCCN1 in nanodiscs.

a, Workflow of the single-particle analysis of MdVCCN1 in nanodiscs. **b**, Representative cryo-EM micrograph of MdVCCN1 in nanodiscs. The red circles indicate individual particles. The white scale bar represents 50 nm. The data collection was performed once. **c**, Local resolution map of MdVCCN1 in nanodiscs. **d**, Angular distribution plot of particles included in the final 3D reconstruction of MdVCCN1 in nanodiscs, with imposed C5 symmetry. **e**, Fourier shell correlation (FSC) curve of the final map of MdVCCN1 in nanodisc. The horizontal dashed line represents the criterion of FSC = 0.143. **f**, Fragmented models of MdVCCN1 in nanodiscs with corresponding cryo-EM density maps.

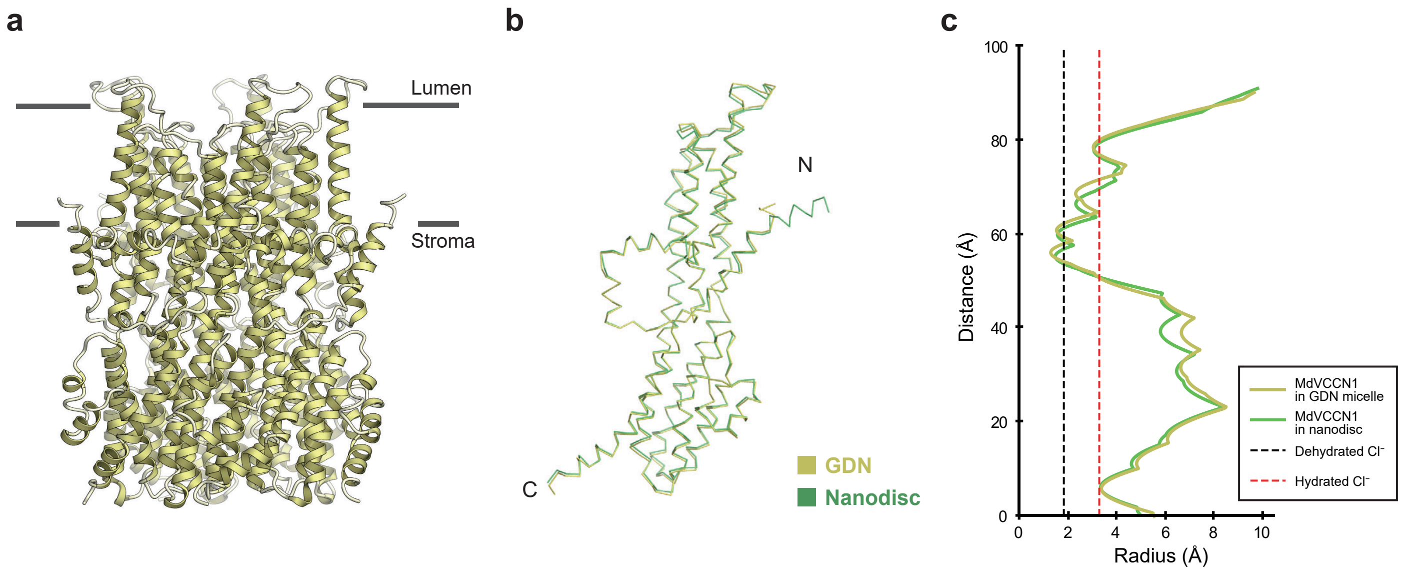

Supplementary Fig. 6 | Structure of MdVCCN1 in GDN.

a, Overall structure of MdVCCN1 in nanodiscs. Gray bars represent the border between the thylakoid membrane and the solution. Predicted topology is indicated for VCCN1, based on the homologous bestrophin channels. **b**, Comparison of the C_α traces of MdVCCN1 in GDN (yellow) and in nanodiscs (green). **c**, Pore radii of MdVCCN1 in GDN (yellow) and in nanodiscs (green).

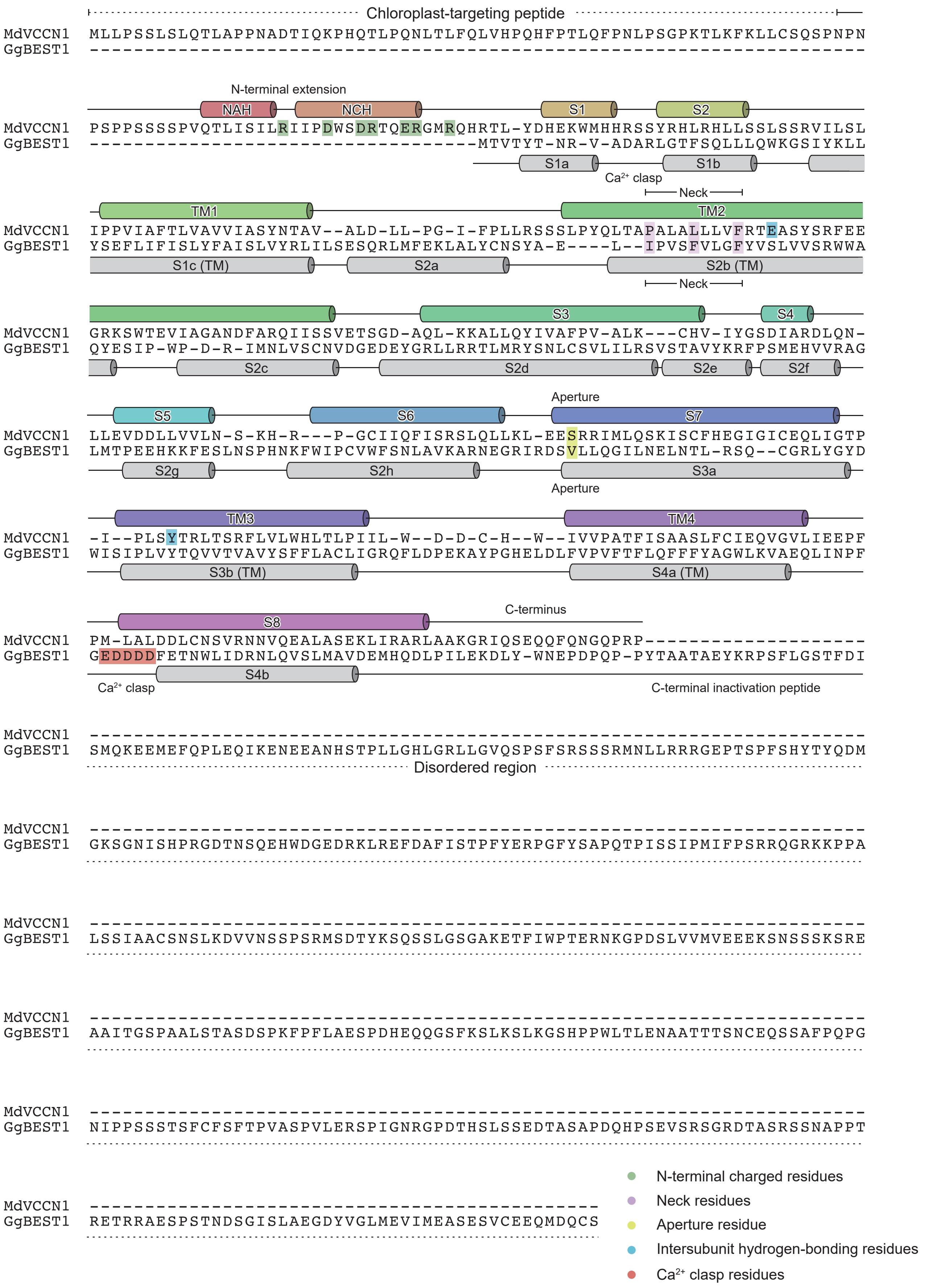

Supplementary Fig. 7 | Sequence alignment of MdVCCN1 and GgBEST1.

Amino acid sequence alignment of MdVCCN1 and *Gallus gallus* bestrophin 1 (GgBEST1 [UniProt: E1C3A0]). The amino acid sequences were aligned using AlignMe[49]. The secondary structures of MdVCCN1 and GgBEST1 are illustrated above and below the sequence, respectively.

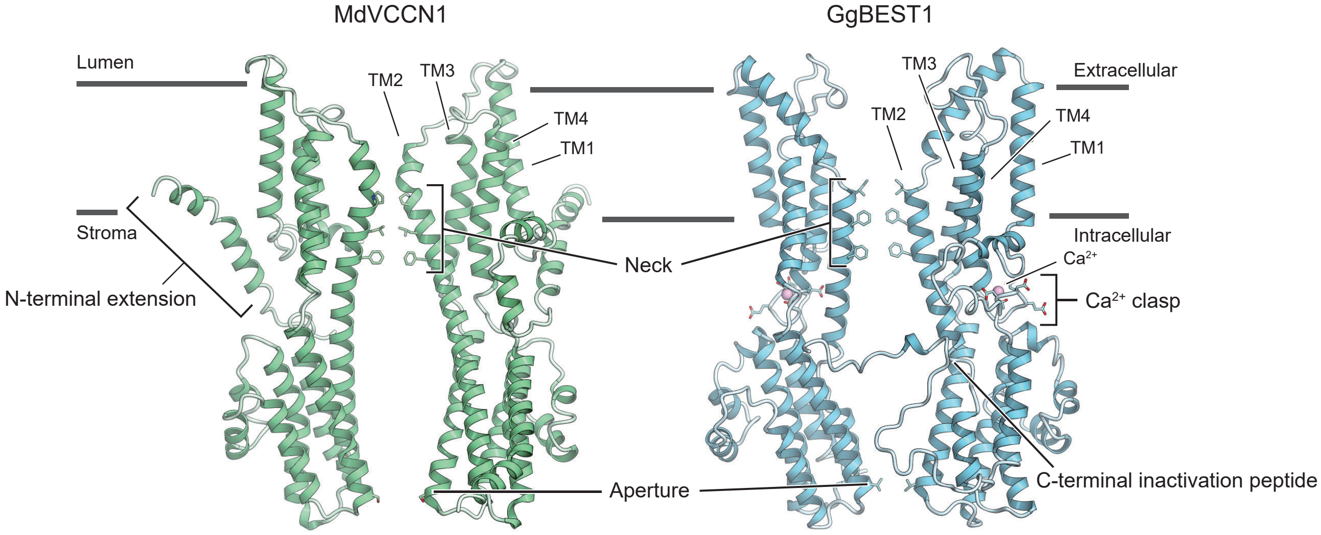

Supplementary Fig. 8 | Structural comparison between MdVCCN1 and GgBEST1.

Structural comparison between MdVCCN1 in nanodiscs and Ca^{2+}-bound closed GgBEST1 (PDB: 6N23). Gray bars represent the border between the membrane and the solution.

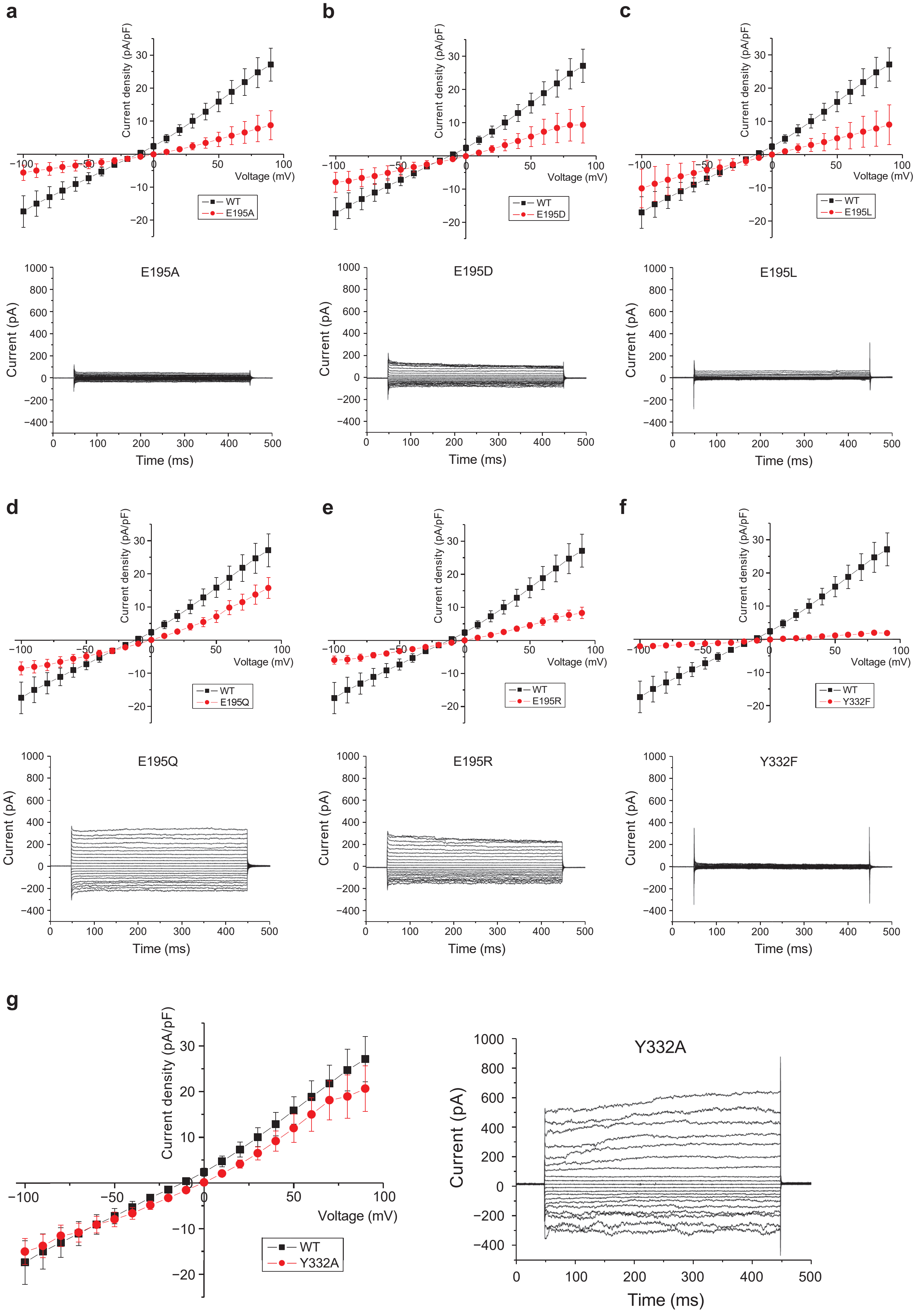

Supplementary Fig. 9 | See next page for caption.

Supplementary Fig. 9 | Electrophysiological assays of Glu195 and Tyr332 mutants.

a-g, The current-voltage relationships in HEK293 cells expressing MdVCCN1 wild-type (black; $n = 16$) and mutants (red; $n = 7$ to 11), and representative traces of MdVCCN1 mutants. See the legend of Fig. 5 for the number of cells used. Error bars represent s.e.m. Source data are provided as a Source Data file.

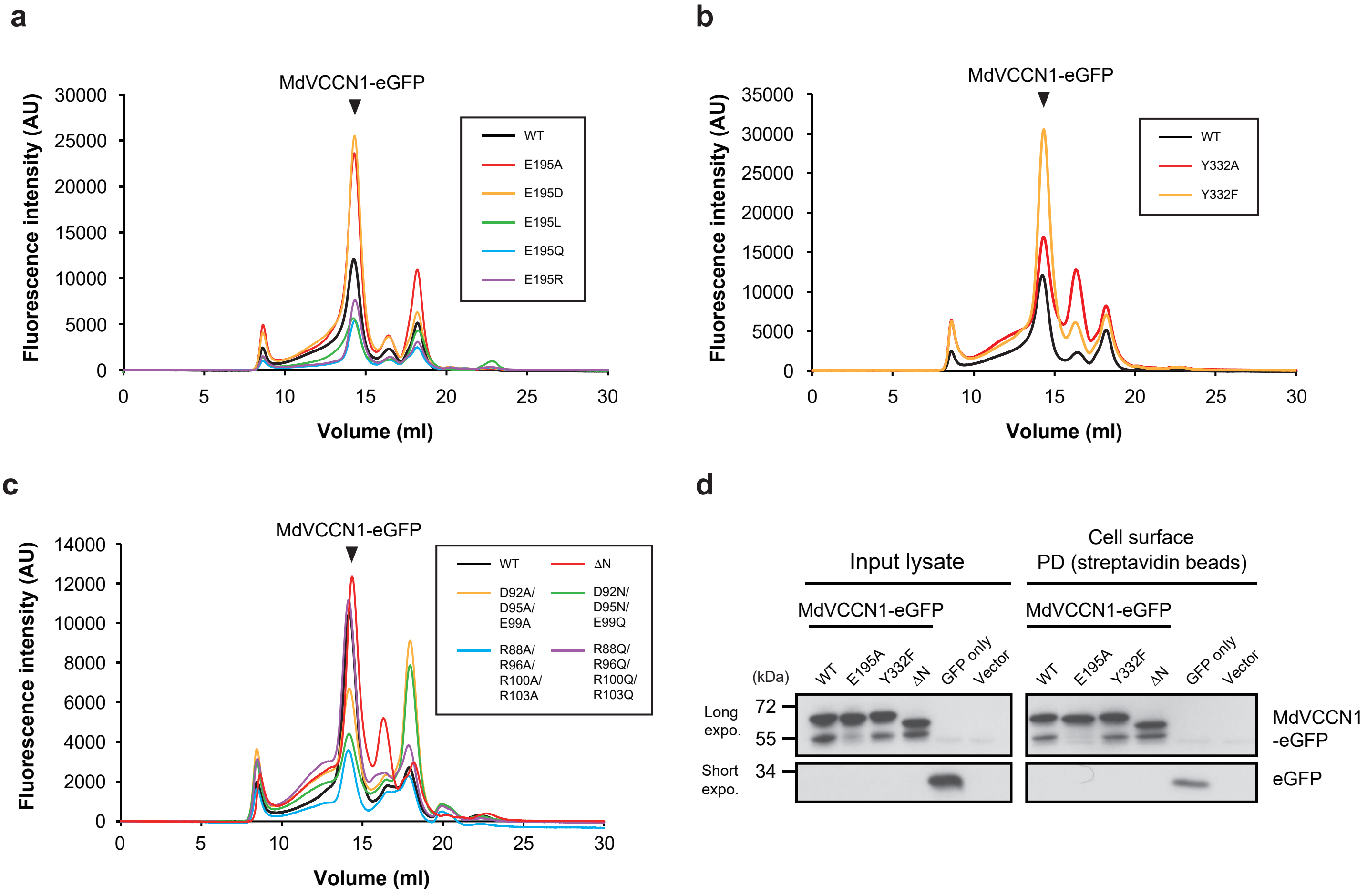

Supplementary Fig. 10 | Expression profile of the N-terminal truncated mutant.

a-**c**, FSEC profiles for the eGFP-fused MdVCCN1, wild-type (black) and mutants: Glu195 mutants (**a**), Tyr332 mutants (**b**), or N-terminal mutants (**c**). All were expressed in HEK293 cells. **d**, Cell surface biotinylation assay for HEK293 cells expressing eGFP-fused MdVCCN1 wild-type, E195A, Y332F, and ΔN mutants, with controls (eGFP only or empty vector transfected HEK cells). The samples were immunoblotted with an anti-GFP antibody. The experiment was performed once.

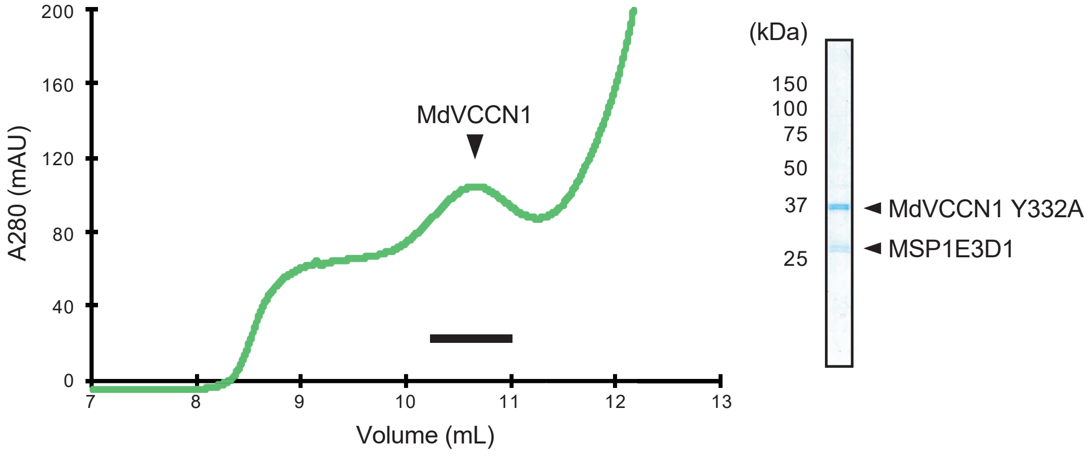

Supplementary Fig. 11 | Purification profiles of MdVCCN1 Y332A.

SEC chromatogram using a Superdex 200 Increase 10/300 column (left) and SDS-PAGE gel image (right) of MdVCCN1 Y332A in nanodiscs. Black bar represents the area of collected fractions. The experiment was performed once.

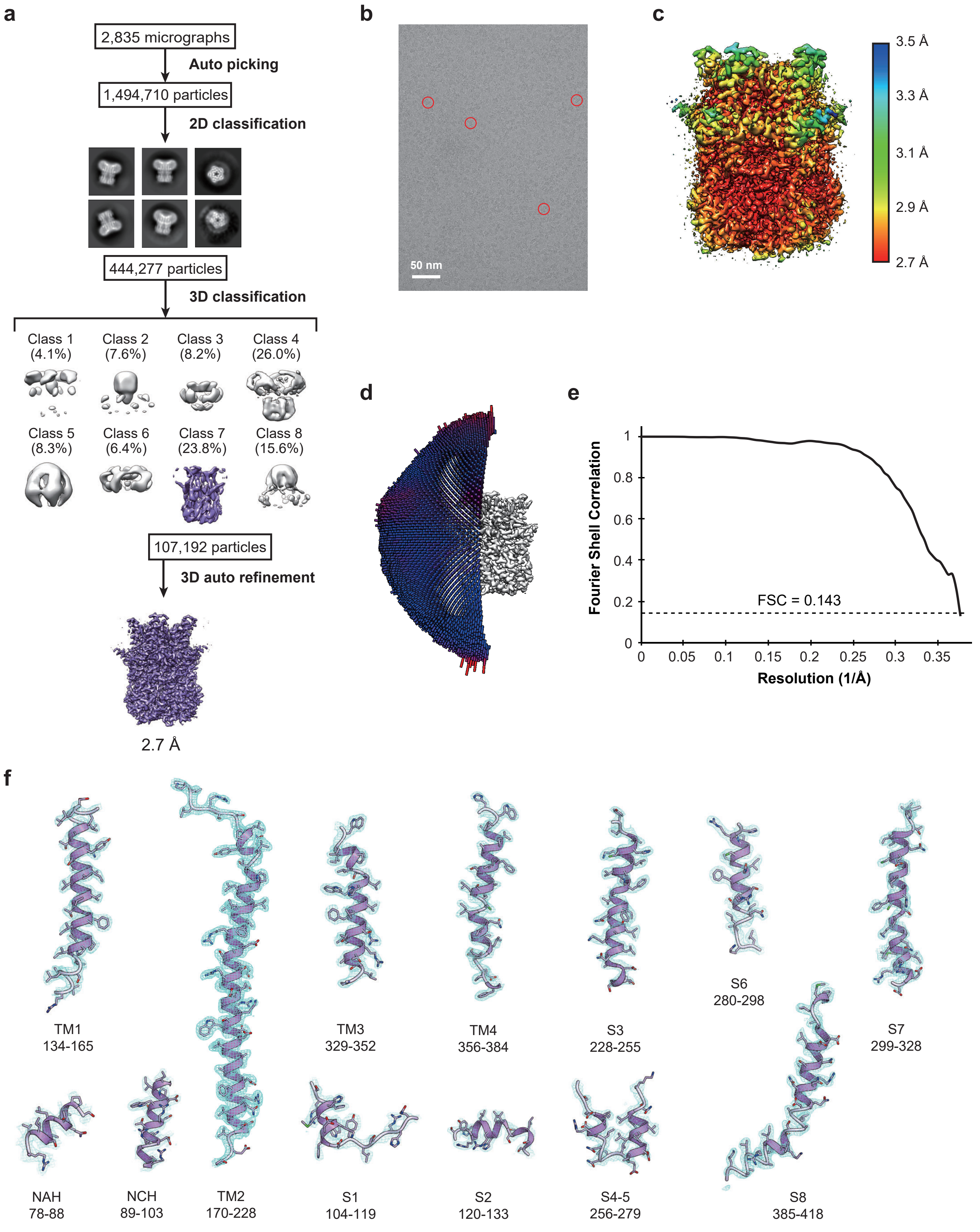

Supplementary Fig. 12 | Single-particle analysis of MdVCCN1 Y332A.

a, Workflow of the single-particle analysis of MdVCCN1 Y332A. **b**, Representative cryo-EM micrograph of MdVCCN1 Y332A. The red circles indicate individual particles. The white scale bar represents 50 nm. The data collection was performed once. **c**, Local resolution map of MdVCCN1 Y332A. **d**, Angular distribution plot of particles included in the final 3D reconstruction of MdVCCN1 Y332A, with imposed C5 symmetry. **e**, Fourier shell correlation (FSC) curve of the final map of MdVCCN1 Y332A. The horizontal dashed line represents the criterion of FSC = 0.143. **f**, Fragmented models of MdVCCN1 Y332A with corresponding cryo-EM density maps.

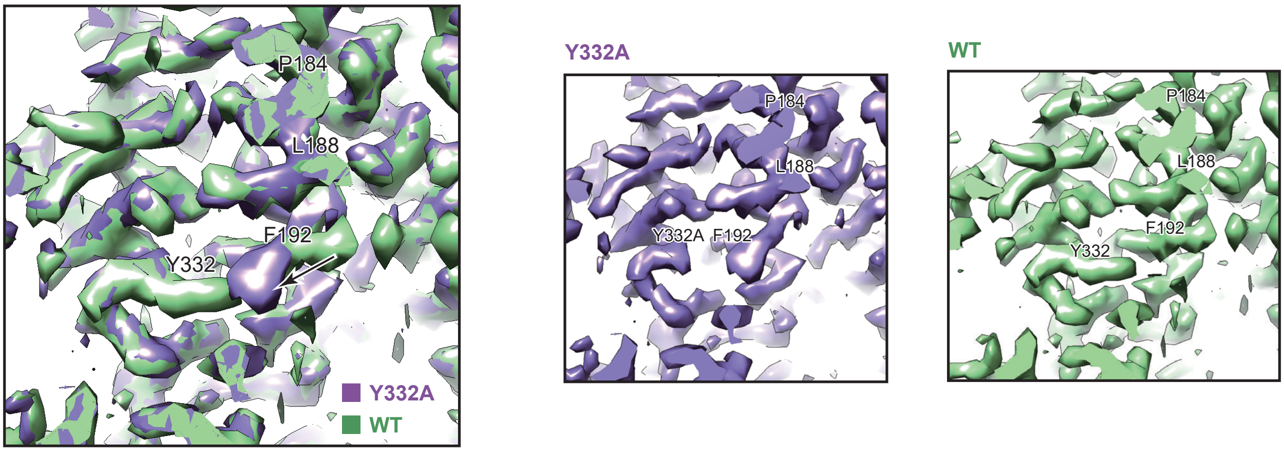

Supplementary Fig. 13 | Conformational change of Phe192 in MdVCCN1 Y332A.

Cryo-EM densities at the neck regions of the MdVCCN1 Y332A mutant (violet) and wild-type (green).

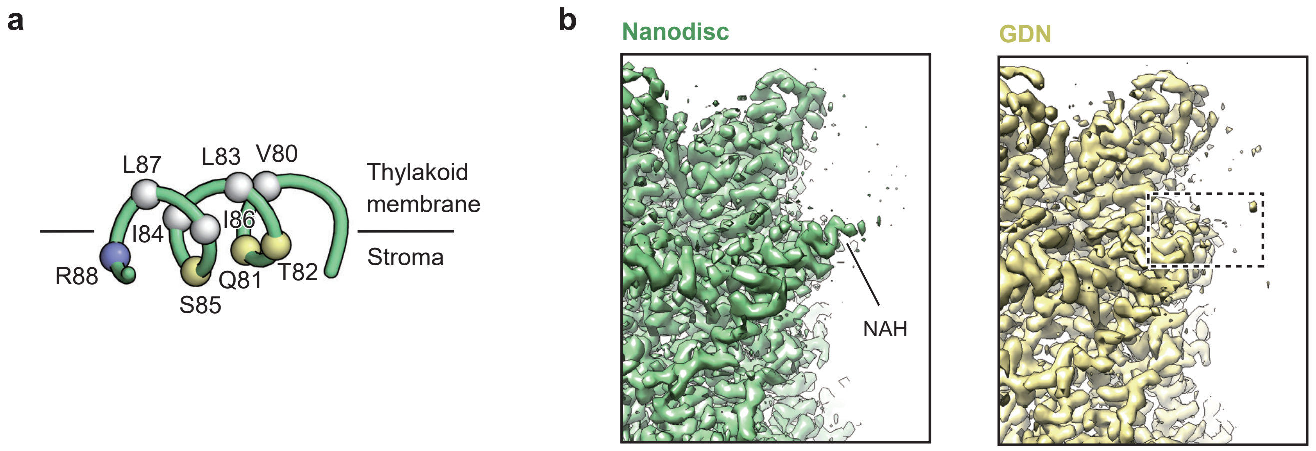

Supplementary Fig. 14 | NAH in lipid environment.

a, Ribbon model of NAH. C_α atoms are represented as balls colored white, yellow, and blue, representing hydrophobic amino acids, uncharged polar amino acids, and positively charged amino acids, respectively. **b**, Cryo-EM densities at the NAH region of MdVCCN1 in nanodiscs (green) and in detergent (yellow).

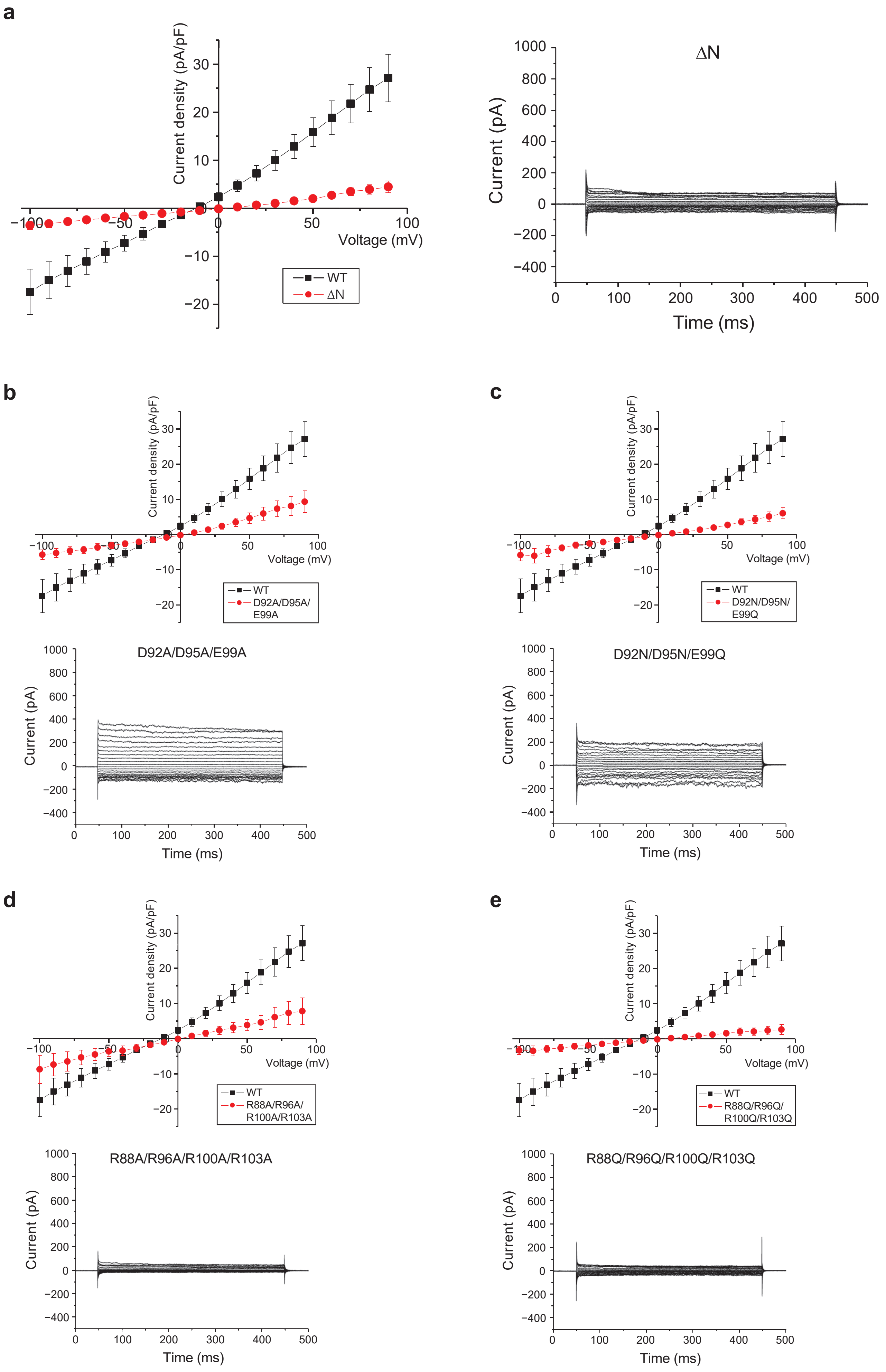

Supplementary Fig. 15 | See next page for caption.

Supplementary Fig. 15 | Electrophysiological assays of N-terminus mutants.

a-e, The current-voltage relationships in HEK293 cells expressing MdVCCN1 wild-type (black; n = 16) and mutants (red; n = 6 to 8), and representative traces of MdVCCN1 mutants. See the legend of Fig. 6 for the number of cells used. Error bars represent s.e.m. Source data are provided as a Source Data file.

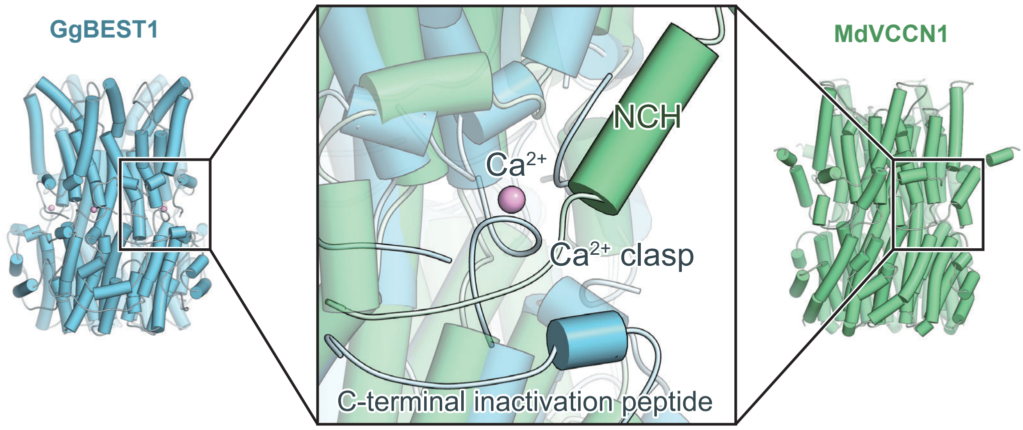

Supplementary Fig. 16 | Structural comparison between MdVCCN1 and GgBEST1.

Structural comparison between the NCH of MdVCCN1 and the structures of GgBEST1 that are involved in the channel regulation. Each is shown as a cartoon model, colored green for MdVCCN1 and blue for GgBEST1.

Supplementary Table 1 | Cryo-EM data collection, model refinement, and validation statistics

	**MdVCCN1 in GDN** (PDB 7EK1, EMDB EMD-31165)	**MdVCCN1 in nanodiscs** (PDB 7EK2, EMDB EMD-31166)	**MdVCCN1 Y332A in nanodiscs** (PDB 7EK3, EMDB EMD-31167)
**Data collection and processing**			
Magnification	×96,000	×105,000	×105,000
Voltage (kV)	300	300	300
Electron exposure (e^{-} $Å^{-2}$)	50	48	48
Defocus range (μm)	−1.0 to −2.5	−0.7 to −1.9	−0.6 to −2.0
Pixel size (Å $pixel^{-1}$)	0.8346	0.83	0.83
Symmetry imposed	C5	C5	C5
Initial particle images (no.)	322,637	730,330	1,494,710
Final particle images (no.)	117,986	345,938	107,192
Map resolution (Å)	3.0	2.7	2.7
FSC threshold	0.143	0.143	0.143
Map resolution range (Å)	2.8-4.2	2.6-3.5	2.6-3.7
Map sharpening *B* factor ($Å^2$)	−127.717	−93.3251	−68.8129
**Refinement**			
Initial model used (PDB code)	4WD8		
Model composition in the asymmetric unit			
Non-hydrogen atoms	2675	2708	2701
Protein residues	336	341	341
R.m.s. deviations			
Bond lengths (Å)	0.0150	0.0131	0.0154
Bond angles (°)	1.8297	1.7284	1.8705
**Validation**			
MolProbity score	1.48	1.10	1.24
Clash score	3.53	3.09	2.36
Poor rotamers (%)	1.34	0.99	1.99
Ramachandran plot			
Favored (%)	96.41	99.12	99.41
Allowed (%)	3.59	0.88	0.59
Outlier (%)	0.00	0.00	0.00

Uncropped scans of gels and blots in Supplementary Figures

Supplementary Fig. 3

a

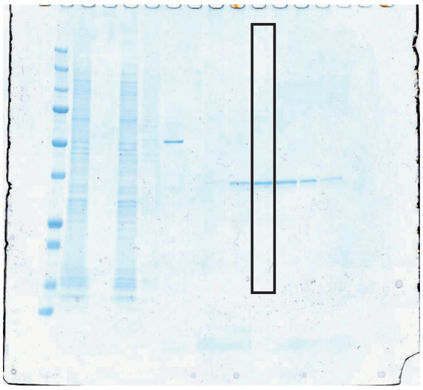

b

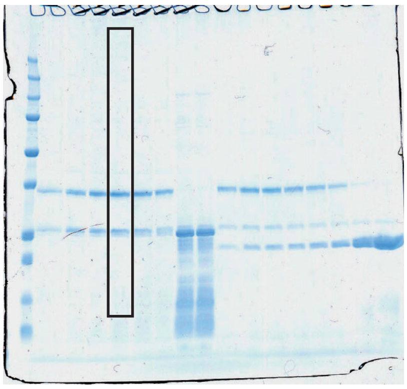

Supplementary Fig. 10d

Long expo.

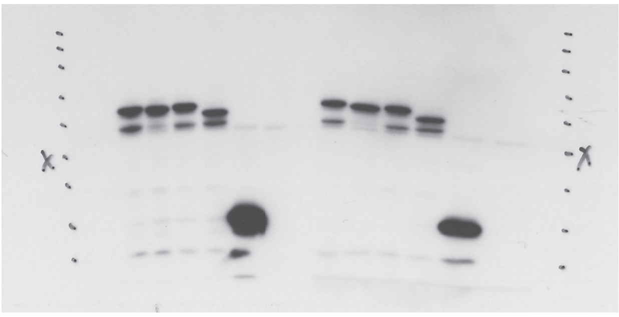

Short expo.

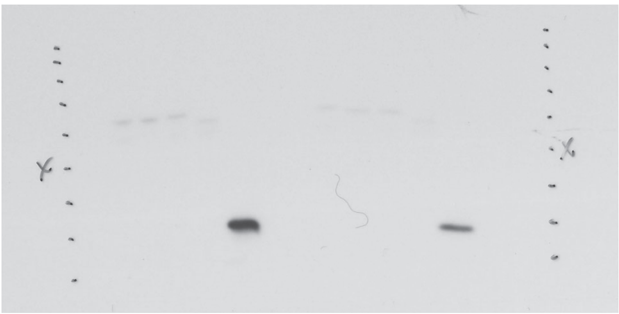

Supplementary Fig. 11

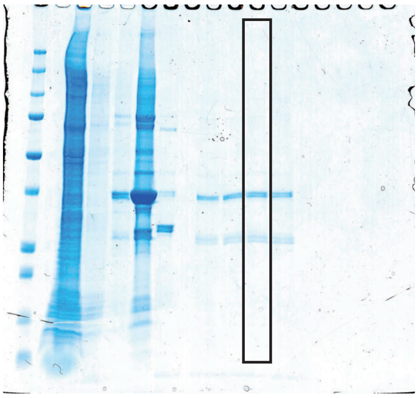

Supplement: Supplementary file 1 — Supplementary Information [file 41467_2022_30292_MOESM1_ESM.pdf]
