# Supplementary material for: Cryo-EM structures of thylakoid-located voltage-dependent chloride channel VCCN1
Source: Nat Commun. 2022 May 6;13:2505. doi: 10.1038/s41467-022-30292-w (PMC9076864; doi:10.1038/s41467-022-30292-w)
Supplement: Supplementary file 3 — Reporting Summary [file 41467_2022_30292_MOESM3_ESM.pdf]

nature research

Corresponding author(s): Osamu Nureki

Last updated by author(s): Apr 4, 2022

Reporting Summary

Nature Research wishes to improve the reproducibility of the work that we publish. This form provides structure for consistency and transparency in reporting. For further information on Nature Research policies, see our Editorial Policies and the Editorial Policy Checklist.

Statistics

For all statistical analyses, confirm that the following items are present in the figure legend, table legend, main text, or Methods section.

n/a	Confirmed	
☐	☒	The exact sample size (*n*) for each experimental group/condition, given as a discrete number and unit of measurement
☐	☒	A statement on whether measurements were taken from distinct samples or whether the same sample was measured repeatedly
☐	☒	The statistical test(s) used AND whether they are one- or two-sided<br>*Only common tests should be described solely by name; describe more complex techniques in the Methods section.*
☒	☐	A description of all covariates tested
☒	☐	A description of any assumptions or corrections, such as tests of normality and adjustment for multiple comparisons
☐	☒	A full description of the statistical parameters including central tendency (e.g. means) or other basic estimates (e.g. regression coefficient) AND variation (e.g. standard deviation) or associated estimates of uncertainty (e.g. confidence intervals)
☐	☒	For null hypothesis testing, the test statistic (e.g. F, t, r) with confidence intervals, effect sizes, degrees of freedom and P value noted<br>*Give P values as exact values whenever suitable.*
☒	☐	For Bayesian analysis, information on the choice of priors and Markov chain Monte Carlo settings
☒	☐	For hierarchical and complex designs, identification of the appropriate level for tests and full reporting of outcomes
☒	☐	Estimates of effect sizes (e.g. Cohen's *d*, Pearson's *r*), indicating how they were calculated

Our web collection on statistics for biologists contains articles on many of the points above.

Software and code

Policy information about availability of computer code

Data collection	EPU 1.5.1.50, SerialEM 3.8.0, SerialEM 3.8.2, pCLAMP10
Data analysis	MotionCor2, CTFFIND 4.1, RELION 3.0, RELION 3.1, HOLE, COOT, PHENIX, UCSF Chimera, CueMol2(http://www.cuemol.org/), Refmac 5, Servalcat, ProSmart, ClustalW, ESPript3, AlignMe

For manuscripts utilizing custom algorithms or software that are central to the research but not yet described in published literature, software must be made available to editors and reviewers. We strongly encourage code deposition in a community repository (e.g. GitHub). See the Nature Research guidelines for submitting code & software for further information.

Data

Policy information about availability of data

All manuscripts must include a data availability statement. This statement should provide the following information, where applicable:

- Accession codes, unique identifiers, or web links for publicly available datasets
- A list of figures that have associated raw data
- A description of any restrictions on data availability

The atomic coordinates have been deposited in the Protein Data Bank (PDB) under the accession numbers 7EK1 [https://doi.org/10.2210/pdb7EK1/pdb] (MdVCCN1 in GDN), 7EK2 [https://doi.org/10.2210/pdb7EK2/pdb] (MdVCCN1 in nanodiscs), and 7EK3 [https://doi.org/10.2210/pdb7EK3/pdb] (MdVCCN1 Y332A in nanodiscs). Cryo-EM density maps have been deposited in the Electron Microscopy Data Bank (EMDB) under the accession numbers EMD-31165 [https://www.ebi.ac.uk/pdbe/entry/emdb/EMD-31165] (MdVCCN1 in GDN), EMD-31166 [https://www.ebi.ac.uk/pdbe/entry/emdb/EMD-31166] (MdVCCN1 in nanodiscs), and EMD-31167 [https://www.ebi.ac.uk/pdbe/entry/emdb/EMD-31167] (MdVCCN1 Y332A in nanodiscs). The source data underlying Figs. 1a-c, 2b, 4d-f, 5b and 6b and Supplementary Figs. 9a-g and 15a-e are provided as a Source Data file with this paper.

Field-specific reporting

Please select the one below that is the best fit for your research. If you are not sure, read the appropriate sections before making your selection.

☒ Life sciences ☐ Behavioural & social sciences ☐ Ecological, evolutionary & environmental sciences

For a reference copy of the document with all sections, see nature.com/documents/nr-reporting-summary-flat.pdf

Life sciences study design

All studies must disclose on these points even when the disclosure is negative.

Sample size	No statistical method was used to determine the sample size. For cryo-EM analyses, sample sizes were determined by the availability of microscope time and the number of particles on electron microscopy grids enough to obtain a structure at the reported resolution. For cell-based analyses, sample sizes were chosen in order to adequately reflect the variance in the measured effect.
Data exclusions	No samples or animals were excluded.
Replication	For cryo-EM analyses, structure determination and related experiments including FSEC, purification, and SDS-PAGE were completed once. For cell-based analyses, all data sets were pooled from at least two independent cells.
Randomization	For cryo-EM analyses, particles were randomly assigned to half-maps for resolution determination following the standard procedures in RELION. For cell-based analyses, randomization was not performed since samples were not divided into two or more groups.
Blinding	For cryo-EM analyses, blinding was not applicable since this type of studies does not use group allocation. For cell-based analyses, blinding was not applied since it was not technically or practically feasible to do so.

Reporting for specific materials, systems and methods

We require information from authors about some types of materials, experimental systems and methods used in many studies. Here, indicate whether each material, system or method listed is relevant to your study. If you are not sure if a list item applies to your research, read the appropriate section before selecting a response.

Materials & experimental systems

n/a	Involved in the study	
☐	☒	Antibodies
☐	☒	Eukaryotic cell lines
☒	☐	Palaeontology and archaeology
☒	☐	Animals and other organisms
☒	☐	Human research participants
☒	☐	Clinical data
☒	☐	Dual use research of concern

Methods

n/a	Involved in the study	
☒	☐	ChIP-seq
☒	☐	Flow cytometry
☒	☐	MRI-based neuroimaging

Antibodies

Antibodies used	Anti-GFP antibody (MBL 598), Anti-GFP antibody (Roche 11814460001), Anti-PsbO antibody (Agrisera AS06-142-33), Anti-Rabbit IgG antibody (MBL458)
Validation	https://ruo.mbl.co.jp/bio/dtl/dtlfiles/598-7-v7.pdf, https://pim-eservices.roche.com/LifeScience/Document/9b6c57f4-1aa2-e911-f08c-f4a30bc86a7b, https://www.agrisera.com/en/artiklar/psbo-33-kda-oec-oxygen-evolving-complex-2.html, https://ruo.mbl.co.jp/bio/dtl/dtlfiles/458-v7.pdf

Eukaryotic cell lines

Policy information about cell lines

Cell line source(s)	Sf9 (ATCC CRL-1711), HEK293S GnTI- (ATCC CRL-3022), HEK293T (ATCC CRL-11268)
Authentication	Cell lines are authenticated by supplier and no further authentication was performed.
Mycoplasma contamination	Not tested.
Commonly misidentified lines (See ICLAC register)	None of commonly misidentified lines were used in this study.
